# Use of Fourier-transform infrared spectroscopy in the diagnosis of rheumatoid arthritis: a pilot study

**DOI:** 10.1007/s11033-016-4079-7

**Published:** 2016-09-17

**Authors:** Lukasz Lechowicz, Magdalena Chrapek, Jozef Gaweda, Mariusz Urbaniak, Iwona Konieczna

**Affiliations:** 1 Department of Microbiology, Institute of Biology, Jan Kochanowski University, Swietokrzyska 15, 25-406 Kielce, Poland; 2 Department of Probability and Statistics, Jan Kochanowski University, Swietokrzyska 11, 25-406 Kielce, Poland; 3 Swietokrzyskie Rheumatology Center, St. Luke Specialized Hospital, Gimnazjalna 41B, 26-200 Konskie, Poland; 4 Organic Chemistry Division, Jan Kochanowski University, Swietokrzyska 11, 25-406 Kielce, Poland

**Keywords:** Diagnosis, Rheumatoid arthritis, Serum, Spectroscopy

## Abstract

Rheumatoid arthritis is an autoimmune inflammatory disease leading to joint cartilage, bone degradation and limitation of mobility. Diagnosis of RA is difficult and complex. There are also no effective methods for clear discrimination between RA patients and non-RA individuals. In this work we use IR spectroscopy to differentiate RA patients and blood donors’ sera. We found differences between investigated sera (RA and non-RA) in range of 3000–2800 and 1800–800 cm^−1^ (W1–W5 regions). Based on mathematical analysis we developed a K-NN model characterized by 85 % of sensitivity and 100 % of specificity. Also we found that, wavenumber 1424 cm^−1^, comprising in W3 region, was the most effective in human sera distinguishing. We conclude that IR spectroscopy may serve as a fast and easy method useful in RA serology.

## Introduction

Rheumatoid arthritis (RA) is one of the common inflammatory disease. It leads to bone and cartilage destruction. Etiology of RA is still unclear, but there is no doubt about its autoimmune background [1]. Early diagnosis is crucial in RA. Presence of autoantibodies in serum is one of the known factors observed in preclinical phase of RA, in some cases even 4 years before disease onset. But their composition may be different and patient dependent [2]. Rheumatoid factor (RFs) and anti-citrullinated peptide antibodies (ACPAs) are the most frequently markers used in disease diagnosis. However, RFs have low specificity [3]. ACPAs are considered as more specific, but repertoire of peptides recognized by ACPAs increase with disease progress. Moreover, before RA symptoms onset, reaction of antibodies may be characteristic also for healthy ACPA-positive individuals [2]. RA patients possess also higher level of osteoprotegerin, soluble receptor activator of nuclear factor kappa B ligand (RANKL) and hyaluronan, as well as chondroitin sulphate in serum, but those molecules are also characteristic for other diseases [4–6].

That, fast and correct diagnosis (sometimes also define of a future disease) is difficult and in clinical practice usually impossible. Spectroscopic analysis, especially infrared spectroscopy (IR), seems to be promising tool in a diagnostic process. IR has been used in the study of biological samples. The IR spectrum of biological samples can be divided into windows correspond to groups of bioorganic compounds: fatty acid (W1 3000–2800 cm^−1^), peptides and proteins (W2 1800–1500 cm^−1^), proteins, phosphate-carrying compounds and fatty acid (W3 1500–1200 cm^−1^), carbohydrates (W4 1200–900 cm^−1^). The fragment W5 (900–750 cm^−1^) corresponds to specific peaks unique for the sample [7, 8].

The aim of this work was to determine the fragments of IR spectra of human sera, characteristic for RA patients.

## Experimental

### Sera

In the experiment 40 sera of blood donors (BD) from Swietokrzyskie Blood Center in Kielce (age average 37.83 ± 14.96), and 29 sera of RA patients (age average 62 ± 13.95) from Swietokrzyskie Rheumathology Center in Konskie were used. Based on age of the individuals, samples were divided into two groups: the young group—50 years old or less (26 blood donors; 5 RA patients); old group—50 years old and more (14 blood donors; 24 RA patients). Sera were stored at −20 °C until measurement of IR spectra. Samples were collected with the approval of the Ethics Committee of the Regional Chamber of Physicians in Kielce.

### The measurement of infrared spectra and its processing

Measurements were made using a Perkin Elmer Spectrum 400 spectrometer in Attenuated Total Reflection (ATR) technique. The IR spectra were measured in range of 3000–750 cm^−1^ with a resolution of 1 cm^−1^. Measurements were performed at a constant temperature and air humidity. The human sera were thawed at room temperature and carefully shaken immediately before the measurement. The sample (1 µl of human serum) was dropped on the crystal spectrometer, and then allowed to stand for 5 min for water evaporate. For each serum three independent replication were made and the average spectrum was calculated. Then, the first derivative was calculated using a five-point stencil:

$${{{f}'}_{k}}\approx \frac{-{{f}_{k+2}}+8\times {{f}_{k+1}}-8\times {{f}_{k-1}}+{{f}_{k-2}}}{12},$$

where ${{{f}'}_{k}}$ is value of 1st derivative for the k wavenumber, ${{f}_{k+2}}$ is the absorbance value of the $k+2$ wavenumber, ${{f}_{k+1}}$ is the absorbance value of the $k+1$ wavenumber, ${{f}_{k-1}}$ is the absorbance value of the $k-1$ wavenumber, ${{f}_{k-2}}$ is the absorbance value of the $k-2$ wavenumber.

The first derivatives were normalized to the range of {0, 1} using the formula:

$${{{V}'}_{k}}=\frac{\left( {{{{f}'}}_{k}}-{{\text{f}}_{min}} \right)}{{{f}_{max}}-{{f}_{min}}}$$

where ${V}_{k}^{\prime}$ is the value of the normalized derivative of the $\mathrm{k}$ wavenumber, ${{{f}'}_{k}}$ is value of the derivative of the k wavenumber, $\left\{ {f_{{min}} ,f_{{max}} } \right\}$ are minimum and maximum values of the first derivative of the spectrum.

The derivatives were used for mathematical analysis. To develop dendrograms we used Manhattan metric and Ward’s method.

### Mathematical model developing for RA patients’ differentiation

For developing of prediction model we used *K* nearest neighbour (K-NN) algorithm. The set of 207 spectra was randomly divided into two subsets: learning subset (157 cases) and validating subset (50 cases). The K-NN model was based on the spectral windows W1–W5. The calculations were performed by using the Statistica 12. The model quality was evaluated on the basis of quality indicators presented in Table 1.

**Table 1:** K-NN model details for RA patients differentiation

Model details	
Number of nearest neighbors	1
Distance	Manhattan
Standardization	No
Averaging	Homogeneous
Quality of the K-NN model	
Total numbers of spectra in validation group	50
True positive	22
False positive	0
False negative	4
True negative	24
Sensitivity	0.85
Miss rate	0.15
Specificity	1.00
Fall-out	0.00
Precision	1.00
False discovery rate	0.00
False omission rate	0.14
Negative predictive value	0.86
Positive likelihood ratio	ND
Negative likelihood ratio	0.15
Diagnostic odds ratio	ND
Accuracy	0.92
Prevalence	0.52

## Results and discussion

IR spectroscopy is very convenient tool in the analysis of biological materials, like tissue sections, cytologic and histologic specimen or biofluids. Types of sample determine methodology of measurement, however there is a manual useful in standardization of the analysis [9].

IR spectroscopy coupled with advanced mathematical analysis have big potential as a screening tool in medical diagnosis. It is a useful method in identification of normal, pre-disease and disease states. Biofluids like blood, serum or plasma seems to be good specimen in regard of many protein biomarkers presence [10]. Lima et al. proved that ATR-FTIR [with genetic algorithm (GA) combined with linear discriminant analysis (LDA)] may be used in early detection of ovarian cancer and differentiation of disease stages [11, 12]. Moreover, ATR-FTIR spectroscopy show higher classification rate than other (Raman) spectroscopic methods [13]. However, most of data are focused on cancer, diabetes or neurodegenerative diseases. Examples of IR spectroscopy usage in RA analysis are limited. We present a pilot study of a differentiation of RA and non-RA sera, based on IR spectra.

### Optimizing the experiment

**Fig. 1:**
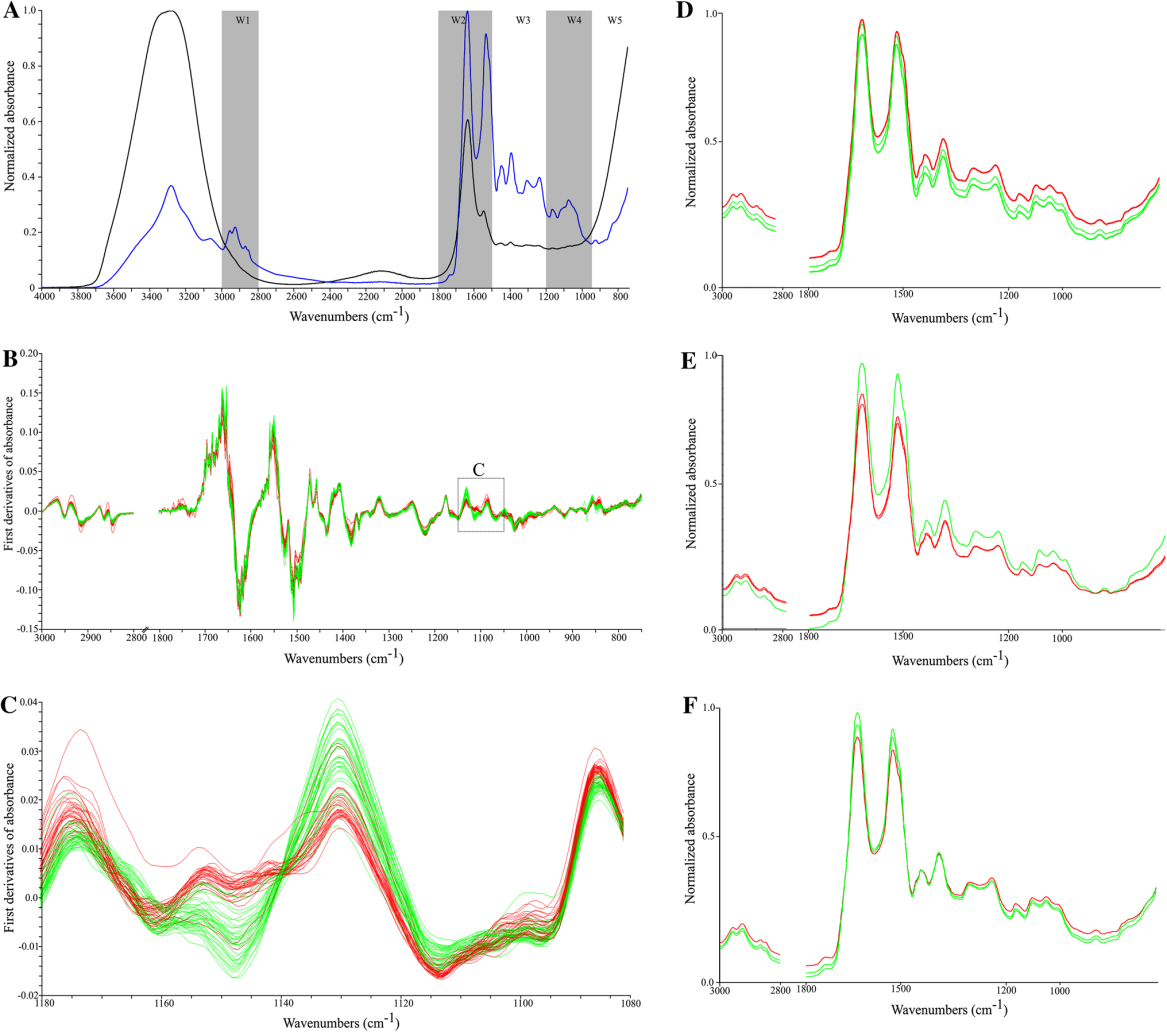
Infrared spectra of human sera. The influence of water content in sample to IR spectrum quality; before and after water evaporation—*black* and *blue* respectively (**a**). First derivatives of patients and a control group spectra (**b**). Fragment of IR spectra most differentiating RA patients and a control group: based on visual observation (**c**)—the *red color* indicates RA patients, while the *green color* indicates the control group. IR spectra misclassified by K-NN model: serum BD.07 (**d**), serum BD.09 (**e**), serum BD.159 (**f**)—the *red color* indicates misclassified spectra, while the *green color* indicates the correctly classified spectra.(Color figure online)

Before performing the proper experiment, a series of measurements were made to determine optimum conditions. We optimized volume and time of drying of investigated samples. The most efficient proved to be the use of 1 µl of human serum, and leaving it to dry for 5 min on the crystal of the apparatus. The use of a larger volume of serum resulted in prolonged water evaporation time and did not result in the improvement of the quality of the IR spectra (data not showed). Presence of water cause reduction of IR light and obscure spectral details [9]. During water evaporation we observed increase of visibility of peaks in different spectrum regions (Fig. 1a). Analogic procedure recommended Baker et al. [9].

### Analysis of IR spectra of human sera

Little data about use of IR spectroscopy in RA serology has been published. Carvalho et al. observed differences between control individuals and RA patients in regions corresponding to proteins, lipids and immunoglobulins (1600–1700 and 1430–1480 cm^−1^) [14]. They used the second derivative of spectra for the mathematical analysis. Khanmohammadi et al. suggested that this range contains medium band of C–N stretching and strong signal due to C–C–N bending of creatinine (1250–1000 and 1230–1100 cm^−1^, respectively) [15].

We also noted some differences between investigated groups in 1600–1700 and 1430–1480 cm^−1^ regions (data not showed). We performed detailed analysis of the first derivative of sera IR spectra. We noted evident distinct discrepancy between RA and blood donors’ groups of human sera in many fragments of the IR spectra. These fragments were uniformly dispersed throughout the analyzed spectra (Fig. 1b) and were found in each of the 5 regions (W1–W5) associated with biological compounds, but in W4 region (range of 1180–1080 cm^−1^) the difference between controls and patients was more clear (Fig. 1c). In this fragment, the shapes of IR spectra for patients and control individuals were different.

### Chemometric analysis

**Fig. 2:**
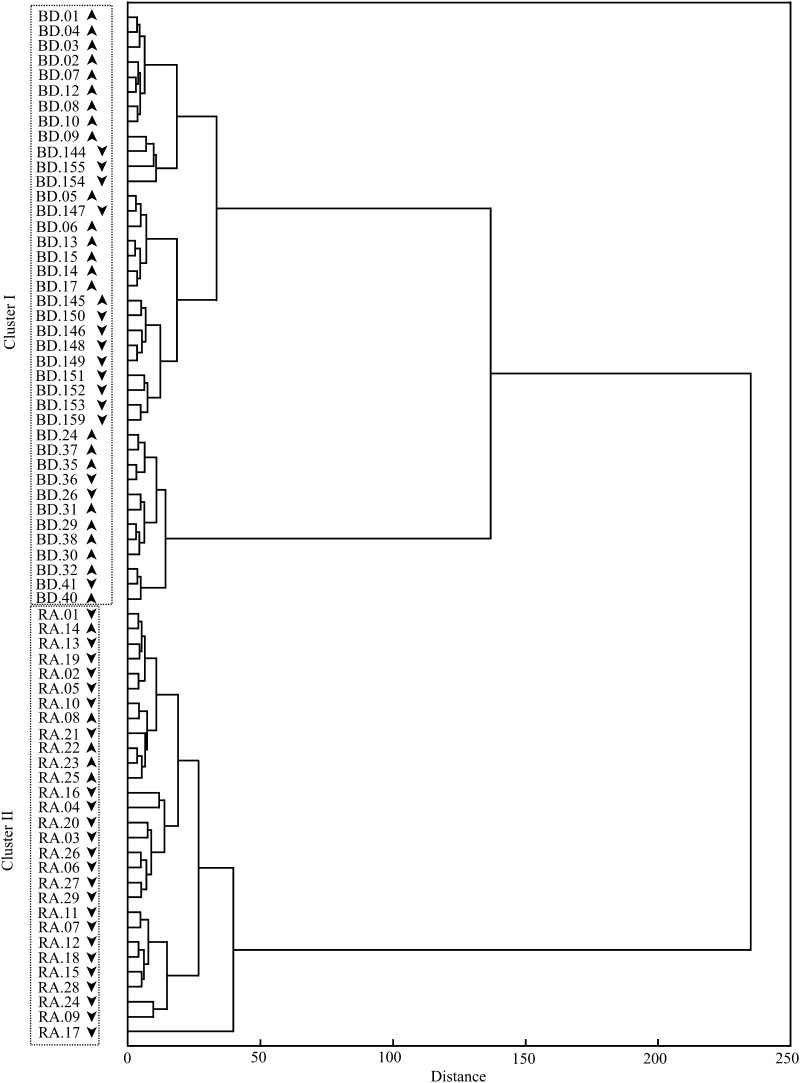
The cluster analysis based on the first derivative of IR spectra of human sera. *Up arrows*—individual younger that 50 years old, *Down arrows*—individuals 50 years old and more. Dendrogram was calculated using Ward`s method and Manhattan length

Cluster analysis (CA), including W1–W5 regions, based on the first derivative of IR spectra revealed the existence of two distinct clusters (Fig. 2). Cluster I contains blood donors, while cluster II contains RA patients. Age (as well as therapy in RA patients, data not shown) has no effect on clustering. The attempts to differentiate RA patients from the control group on the basis of each region separately were unsuccessful (data not shown).

Because of this reason the whole IR spectra range (W1–W5) for developing a predictive mathematical model (the K nearest neighbor algorithm) for discriminating RA patients and blood donors has been used. Our model is characterized by moderate sensitivity (85 %) and very high specificity (100 %)—Table 1. All incorrect classified IR spectra sera belong to blood donors (Fig. 1d–f). The incorrectly classified IR spectra (DB09 and BD159) are characterized by a lower absorbance band of amide I and amide II. Most likely, the water content in the sera was too high and resulting absorbance decrease in the entire range of the spectra. In case of one serum (BD07) higher absorbance has been observed and reason for such discrepancy in measurements is unknown. Nevertheless, the proposed model is a promising tool for detecting rheumatoid arthritis based on IR spectra of sera.

Regardless of this model we also noted that one wavenumber in IR spectra of analyzed sera is also useful in samples differentiation. The average values of wavenumber 1424 cm^−1^ (W3 region) differ significantly (p < 0.0001, *t*-test) in RA patients and control group. For this value distribution in both groups was normal (p = 0.58 for RA patients and p = 0.35 for blood donors) based on Shapiro–Wilk test.

Differentiating between RA patients and control group was created by following rule: if the value of the first derivative of 1424 cm^−1^ is <0.013115, then the person is RA patient; otherwise person does not suffer from RA.

Value 1424 cm^−1^ comprise in mixed region W3 carrying peaks of proteins, phosphate molecules and fatty acid. It was shown that this wavenumber is connected with H–C–H and O–C–H in-plane bending vibration characteristic for proteins (especially for two amino acids: proline and tryptophan) [16, 17]. Proline is one of amino acid which occurs in collagen type I and hydroxyproline is a marker of bone collagen degradation [18]. On the other hand, increased level of tryptophan was noted for RA patients [19]. However, using only one wavenumber for differentiating of sera samples is discussing. In such cases there is a risk of loose of efficacy when investigated group is changed. Much more proper seems to be model taking into account a whole IR spectrum range (W1–W5).

## Conclusion

RA is one of the most common systemic diseases leading to joint deformation and increased mortality. It concerns up to 1 % of world population, mostly women. Early and precise diagnosis of RA is crucial. However, diagnostic tests are still insufficient (conventional disease markers are present also in healthy population or are not specific only for RA) [14].

In this work we used ATR-FTIR technique to differentiate human sera of RA patients and non-RA blood donors. Our results show, that FTIR spectroscopy may be promising tool in RA investigation. It may serve as a fast, low cost and sensitive sera discriminatory method or as a prognostic test.

## References

[1] Terato K, Do C, Shionoya H (2015). Slipping through the cracks: linking low immune function and intestinal bacterial imbalance to the etiology of rheumatoid arthritis. Autoimmune Dis.

[2] Schaeverbekea T, Truchetet M, Richez C (2012). When and where does rheumatoid arthritis begin?. Joint Bone Spine.

[3] Bax M, Huizinga T, Toes R (2014). The pathogenic potential of autoreactive antibodies in rheumatoid arthritis. Semin Immunopathol.

[4] Kolarz G, Schödl C, Skoumal M, Woloszczuk W, Wottawa A (2003). Osteoprotegerin serum levels in rheumatoid arthritis. Journal für Mineralstoffwechsel & Muskuloskelettale Erkrankungen.

[5] Pothacharoen P, Teekachunhatean S, Louthrenoo W, Yingsung W, Ong-Chai S, Hardingham T, Kongtawelert P (2006). Raised chondroitin sulfate epitopes and hyaluronan in serum from rheumatoid arthritis and osteoarthritis patients. Osteoarthr Cartil.

[6] Bezerra MC, Carvalho JF, Prokopowitsch AS, Pereira RMR (2005). RANK, RANKL and osteoprotegerin in arthritic bone loss. Braz J Med Biol Res.

[7] Naumann D, Helm D, Labischinski H, Giesbrecht P, Nelson W (1991). The characterization of microorganisms by Fourier-transform infrared spectroscopy (FT-IR). Modern techniques for rapid microbiological analysis.

[8] Naumann D, Meyers R, Infrared Spectroscopy in Microbiology (2000). Encyclopedia of analytical chemistry.

[9] Baker MJ, Trevisan J, Bassan P, Bhargava R, Butler HJ, Dorling KM, Fielden PR, Fogarty SW, Fullwood NJ, Heys KA, Hughes C, Lasch P, Martin-Hirsch PL, Obinaju B, Sockalingum GD, Sulé-Suso J, Strong RJ, Walsh MJ, Wood BR, Gardner P, Martin FL (2014). Using Fourier transform IR spectroscopy to analyze biological materials. Nat Protoc.

[10] Mitchell AL, Gajjar KB, Theophilou G, Martin FL, Martin-Hirsch PL (2014). Vibrational spectroscopy of biofluids for disease screening or diagnosis: translation from the laboratory to a clinical setting. J Biophotonics.

[11] Gajjar K, Trevisan J, Owens G, Keating PJ, Wood NJ, Stringfellow HF, Martin-Hirsch PL, Martin FL (2013). Fourier-transform infrared spectroscopy coupled with a classification machine for the analysis of blood plasma or serum: a novel diagnostic approach for ovarian cancer. Analyst.

[12] Lima KMG, Gajjar KB, Martin-Hirsch PL, Martin FL (2015). Segregation of ovarian cancer stage exploiting spectral biomarkers derived from blood plasma or serum analysis: ATR-FTIR spectroscopy coupled with variable selection methods. Biotechnol Prog.

[13] Owens GL, Gajjar K, Trevisan J, Fogarty SW, Taylor SE, Da Gama-Rose B, Martin-Hirsch PL, Martin FL (2014). Vibrational biospectroscopy coupled with multivariate analysis extracts potentially diagnostic features in blood plasma/serum of ovarian cancer patients. J Biophotonics.

[14] Carvalho C, Silva AC, Santosa T, Martina A, Fernandesa AC, Andradeb LE, Raniero L. (2012) A rheumatoid arthritis study by Fourier transform infrared spectroscopy. In: Mahadevan-Jansen A, Petrich W (eds) Biomedical vibrational spectroscopy V: advances in research and industry. doi:10.1117/12.907117

[15] Khanmohammadi M, Ghasemi K, Garmarudi AB, Ramin M (2015). Diagnostic prediction of renal failure from blood serum analysis by FTIR spectrometry and chemometrics. Spectrochim Acta A Mol Biomol Spectrosc.

[16] Fan M, Dai D, Biao H, Salih S (2012). Fourier transform infrared spectroscopy for natural fibres. Fourier transform—materials analysis.

[17] Barth A, Zscherp C (2002). What vibrations tell us about proteins. Q Rev Biophys.

[18] Seibel M (2005). Biochemical markers of bone turnover part I: biochemistry and variability. Clin Biochem Rev.

[19] Kim S, Hwang J, Xuan J, Jung YH, Cha HS, Kim KH (2014). Global metabolite profiling of synovial fluid for the specific diagnosis of rheumatoid arthritis from other inflammatory arthritis. PloS One.

